# Novel manufacturing process of pneumococcal capsular polysaccharides using advanced sterilization methods

**DOI:** 10.3389/fbioe.2024.1451881

**Published:** 2024-08-07

**Authors:** Yuelong Li, Xin Cao, Xueting Huang, Yanli Liu, Jianlong Wang, Qian Jin, Jiankai Liu, Jing-Ren Zhang, Haifa Zheng

**Affiliations:** ^1^ Beijing Minhai Biotechnology Co. Ltd., Beijing, China; ^2^ Center for Infection Biology, School of Medicine, Tsinghua University, Beijing, China

**Keywords:** capsular polysaccharide, sterilization agent, purification, impurity, phase variation

## Abstract

Pneumococcal disease is caused by *Streptococcus pneumoniae*, including pneumonia, meningitis and sepsis. Capsular polysaccharides (CPSs) have been shown as effective antigens to stimulate protective immunity against pneumococcal disease. A major step in the production of pneumococcal vaccines is to prepare CPSs that meet strict quality standards in immunogenicity and safety. The major impurities come from bacterial proteins, nucleic acids and cell wall polysaccharides. Traditionally, the impurity level of refined CPSs is reduced by optimization of purification process. In this study, we investigated new aeration strategy and advanced sterilization methods by formaldehyde or β-propiolactone (BPL) to increase the amount of soluble polysaccharide in fermentation supernatant and to prevent bacterial lysis during inactivation. Furthermore, we developed a simplified process for the CPS purification, which involves ultrafiltration and diafiltration, followed by acid and alcohol precipitation, and finally diafiltration and lyophilization to obtain pure polysaccharide. The CPSs prepared from formaldehyde and BPL sterilization contained significantly lower level of residual impurities compared to the refined CPSs obtained from traditional deoxycholate sterilization. Finally, we showed that this novel approach of CPS preparation can be scaled up for polysaccharide vaccine production.

## 1 Introduction

*Streptococcus pneumoniae* is lancet-shaped, Gram-positive and facultative anaerobic bacteria with more than 100 known serotypes, causing various infections, such as pneumonia, meningitis, otitis media, and bacteremia, with children under 5 years old being the most susceptible population (Kadioglu et al., 2008; Weiser et al., 2018; Paton and Trappetti, 2019). As antibiotic resistance becomes increasingly more prevalent, prevention of pneumococcal disease by vaccination has become more urgent (Lees et al., 2017; Weiser et al., 2018; Fitzgerald and Waterer, 2019; Guo and Qiao, 2021; Al-Jumaili et al., 2023).

Pathogenic factors of *S*. *pneumoniae* include polysaccharide capsule, hemolysins, and surface proteins, among which the capsule is the most important virulence factor (Kadioglu et al., 2008; Geno et al., 2015; Paton and Trappetti, 2019). Capsular polysaccharides (CPSs) on bacterial surface not only protect the bacteria from being cleared by mucus in the nasopharynx, but also shield bacterial surface structures from recognition by the host innate immune system (Hyams et al., 2010; Hamaguchi et al., 2018). In particular, the capsule enables pneumococci to bypass phagocytic killing by the liver resident macrophages during blood infection (An et al., 2022; Huang et al., 2022). Pneumococcal CPSs are also effective vaccine antigens that stimulate the host to produce protective antibodies (Avery et al., 1925; Avery and Heidelberger, 1925; Whitney et al., 2006; Fusco et al., 2007). Hence, the CPSs of prevalent serotypes have been formulated into various forms of pneumococcal vaccines (Jones and Currie, 1991; Talaga et al., 2002). Purified CPS antigen in pneumococcal vaccines must meet strict quality standards, including high immunogenicity and adequate safety. Quality control requirements related to vaccine safety include residual proteins, nucleic acids, pyrogenic substances, and chemical reagents associated with purification (Jones and Currie, 1991; Talaga et al., 2002; Jones, 2015; Morais et al., 2018; Lee et al., 2020).

Because pneumococcal CPSs are attached to the cell wall (Larson and Yother, 2017), in the final stage of fermentation, sodium deoxycholate (DOC) or other inactivating agents generally are added to the fermentation broth to inactivate the bacteria by inducing the autolysis, which is followed by harvesting the fermentation lysate for the recovery and purification of CPSs. This approach typically leads to the release of a large amount of cellular debris, proteins, nucleic acids, cell wall polysaccharides and other cellular components (Morais et al., 2018; Lee et al., 2020; Gaikwad et al., 2021). Due to the physical and chemical properties of soluble proteins, removing residual proteins is a major challenge in obtaining CPSs with adequate purity, especially for serotypes 5, 10A, 14, etc. The mainstream purification techniques include the following (Morais et al., 2018):

Removal of impurities by precipitation is an important method. For instance, Cano et al. disclosed methods for purifying CPSs involving one or two alcohol precipitations, CTAB precipitation as well as carbon absorption (Cano et al., 1980). Flocculants were also used to precipitate cell debris, host cell proteins and nucleic acids, these flocculants include aluminum phosphate (Macha et al., 2014), alum (Liangjia et al., 2021) and silica particles (Venkat et al., 2022). Acid precipitation is also an important technique to precipitate and remove proteins at low pH, followed by activated carbon filtration (Yuan et al., 2008), alkaline treatment (Bahler et al., 2008), consecutive ethanol precipitations (Jung et al., 2011; Xiaobo et al., 2016; Libo et al., 2017) and CTAB precipitations (Lee et al., 2020). Generally, purification through precipitation is achieved by a complex purification process, which involves many labor-intensive and technically demanding steps. Meanwhile, novel methods involving a single or a combination of hydrophobic interaction chromatography, ion exchange or size exclusion chromatography have been developed for obtaining highly purified CPSs (Suárez et al., 2001; Kapre et al., 2012; Yongjie et al., 2014; Zanardo et al., 2016; Kapre Subhash and Datta Anup, 2020; Venkat et al., 2022). However, chromatography techniques are typically time-consuming, expensive and result in low recovery yield. In addition, enzymatic method remains an important technique for polysaccharide purification (Gonçalves et al., 2003; Jiandong et al., 2015; Keming et al., 2015). However, this purification technique requires the removal of exogenous enzymes through multiple methods and final validation of removal efficiency.

The optimization of the CPS purification process aims to remove impurities more efficiently and is an important aspect for reducing the residual impurities in purified polysaccharides. In the meantime, the optimization of the fermentation and inactivation process of *S*. *pneumoniae* could hopefully reduce the generation of impurities in fermentation broth after inactivation of pneumococcus, thereby simplifying the purification process of pneumococcal CPSs. Gonçalves et al. adjusted the cultivation gas environment of fermentation in their studies, demonstrated that a large amount of CPSs shed from the surface and entered the fermentation supernatant, it was therefore proposed that by extracting only the CPSs from the fermentation supernatant, the complexity of purification process hopefully could be significantly reduced (Gonçalves et al., 2002; Gonçalves et al., 2006). Taddese et al. (2021) showed that BPL, ethanol and formalin treatments generated inactivated bacteria with preserved cellular integrity. Gonçalves et al. (2014) applied BPL for the preparation of inactivated pneumococcal whole cell vaccine. Richard et al. (2016) also proposed using BPL to inactivate pneumococcal cells, since BPL can effectively release soluble polysaccharides from the surface of pneumococcus while maintaining the overall structure of the pneumococcal cells. Moreover, BPL is easily hydrolyzed, eliminating the need for a specialized removal step. Zanardo et al. (2016) applied thiomersal to inactivate the bacteria and removed intact pneumococcal cells by filtration, which prevented the release of a large number of intracellular contaminants.

Therefore, in this study we firstly integrated novel aeration strategy and inactivation with formaldehyde and BPL for fermentation broth, which can efficiently increase the amount of soluble polysaccharide in fermentation supernatant and prevent bacterial lysis during inactivation, in the meantime we developed a simplified downstream purification process to obtain refined polysaccharide and finally compared the test results of impurities for refined polysaccharide with three different inactivation methods (DOC, BPL and formaldehyde). This novel fermentation, inactivation and purification process were also conducted on pilot scale for verification of process scalability.

## 2 Materials and methods

### 2.1 Bacterial strains

*S*. *pneumoniae* serotype 5 (CMCC 31457), 7F (CMCC 31510), 10A (CMCC 31218), 14 (CMCC 31608), and 15B (CMCC 31638) strains were purchased from National Center for Medical Culture Collections, and the lyophilized strain was used to establish master seed lot and working seed lot, which were identified and tests were conducted to verify the characteristics of the strain by the National Institutes for Food and Drug Control of China.

### 2.2 Reagents and equipments

#### 2.2.1 Reagents

Solid Medium: Soy peptone (Kerry), L-Cysteine hydrochloride (Tianjin Pharmaceutical Group Co., Ltd.), Tryptophan (Sinopharm Chemical Reagent Co., Ltd.), Tyrosine (Tianjin Pharmaceutical Group Co., Ltd.), Dipotassium hydrogen phosphate (Sinopharm Chemical Reagent Co., Ltd.), Hydrochloric acid (Hunan Erkang Pharmaceutical Co., Ltd.), Agar powder (Beijing Aoboxing Biotechnology Co., Ltd.) etc.

Liquid Medium: Biotin, Vitamin B2, Vitamin B6, Vitamin B1, Adenine, Uracil (SIGMA-ALDRICH); Glutamine (Hebei Bailingwei Ultrafine Materials Co., Ltd.), L-Asparagine (Sinopharm Chemical Reagent Co., Ltd.), Choline chloride, Magnesium sulfate heptahydrate, Ferrous sulfate heptahydrate, Zinc sulfate heptahydrate (Sinopharm Chemical Reagent Co., Ltd.), Thioglycolic acid (Sinopharm Chemical Reagent Co., Ltd.), Catalase (SIGMA-ALDRICH), Soy peptone (Kerry), Glucose (Shandong Saint-Show Technology and Pharmaceutical Co., Ltd.), Sodium hydroxide (Chengdu Huayu Pharmaceutical Excipients Co., Ltd.) etc.

Inactivation agent: DOC (SIGMA-ALDRICH), formaldehyde (SIGMA-ALDRICH) or β-propiolactone (Ferak Berlin).

Purification reagent: Acetic acid (Chengdu Huayu Pharmaceutical Excipients Co., Ltd.), Disodium hydrogen phosphate (Hunan Jiudian Hongyang Pharmaceutical Co., Ltd.), Sodium dihydrogen phosphate (Hunan Jiudian Hongyang Pharmaceutical Co., Ltd.), Sodium chloride (Hubei Wuhuan Salt Industry Group Co., Ltd.), Anhydrous sodium acetate (Nanjing Chemical Reagent Co., Ltd.), Calcium chloride (Beijing Yanjing Pharmaceutical Co., Ltd.), Sodium hydroxide (Chengdu Huayu Pharmaceutical Excipients Co., Ltd.), Anhydrous ethanol (Nanjing Chemical Reagent Co., Ltd.) etc.

#### 2.2.2 Equipments

Fermentation: 15 L Microbial Bioreactor (Shanghai Bailun Biotechnology), CO_2_ Incubator (Thermo Fisher), UV-Visible Spectrophotometer (Beijing Puxi Technology), Biological safety cabinet (Suzhou Antai Air Technology), XB43 Microscope (Olympus), Bioprocess Analyzer (Shenzhen Hillman Biotechnology), Vacuum Freeze Dryer (Shanghai Dongfulong), Biochemical Incubator (Qingdao Haier Biomedical Co., Ltd.), High-Speed Centrifuge (Thermo Fisher).

Purification: Magnetic Stirrer (Shanghai Meiyingpu Instrument Manufacturing Co., Ltd.), Electronic Balance (Mettler Toledo), Electronic Platform Scale (Mettler Toledo), Multiparameter Tester (Mettler Toledo) and high speed centrifuges were purchased from Thermo fisher; The ultrafiltration membranes were purchased from Millipore.

### 2.3 Bioreactor fermentation

Frozen stock culture (1 mL) from working seed lot was used to inoculate solid medium and cultured in a carbon dioxide incubator for 9–15 h (with a carbon dioxide concentration of 10% and a cultivation temperature of 36°C); the strain was then inoculated to 1 L flask containing 300 mL of liquid medium, and the flasks were incubated at 36°C and 90 rpm to obtain an optical density (OD) of 0.4–0.6 at 600 nm; It was passaged to next liquid cultivation under the same conditions with higher volume. When the OD_600_ of the culture reached 0.4–0.6, it was transferred at an inoculation volume of 4%–7% into bioreactors containing fermentation medium.

Laboratory scale fed-batch fermentations were carried out in 15-L bioreactor (Shanghai Bailun Biotechnology, China) with a working volume of 10 L; Pilot scale fed-batch fermentations were carried out in 300-L bioreactor (Shanghai Gjbio tec, China) with a working volume of 200 L. Bioreactor cultivations were conducted under 100% carbon dioxide atmosphere (0.1 vvm), at 36°C and the pH was controlled at 7.2 by addition of 5 M NaOH. The agitation speed was maintained using a mechanically driven impeller (90 rpm). During the fermentation process, a continuous feed medium was used to maintain the glucose concentration above 5 g/L in the fermentation system. In the late stage of the logarithmic growth phase of fermentation, the cultivation was switched from anaerobic environment to air sparging, which promoted the release of CPSs from the surface of pneumococcus into cultivation medium. At various intervals of time during cultivation, samples were taken for the measurement of optical density at 600 nm, analysis of glucose, polysaccharide in the supernatant and total polysaccharide (DOC was added into samples to lyse the cells and samples were centrifuged at 13,000 g, 15 min, 4°C to obtain pellets and supernatants).

### 2.4 Inactivation treatment

In the final stage of fermentation, inactivation reagents were added for sterilization. The fermentation broth was treated with 0.1% DOC, 0.1% BPL and 1% formaldehyde and stirred for 40 min, then incubated at 4°C for 12 h for sterilization. The cell suspension was plated on blood agar plates enriched with brain heart infusion (BD/Difco). The plates were incubated in 10% CO_2_ at 36°C for up to 3 days and the colony forming units were recorded.

Inactivated pneumococcal cells were centrifuged with the lowest possible g-value to achieve a fluffy bacterial pellet and resuspended in the fluffy bacteria pellet in fixation solution, the bacteria pellet was washed and fixed several times to obtain the final bacterial pellet, samples were prepared and tested according to Sven and Manfred (Hammerschmidt and Rohde, 2019).

### 2.5 Purification of CPSs

A simplified purification process was employed, which involves ultrafiltration and diafiltration to remove small molecular impurities, followed by acid and alcohol precipitation to eliminate protein and nucleic acid impurities, and finally diafiltration and lyophilization to obtain purified polysaccharide.

1. After sterilization, the fermentation broth was centrifuged at 8,000 rpm for 30 min to collect the supernatant, which was ultrafiltered using 100 kDa membrane to obtain concentrated ultrafiltrate, diafiltration to remove small molecules and impurities.
2. Acetic acid was added to the aforementioned concentrate to adjust the pH to 4.00, after thorough mixing, it was left to stand at 2°C–8°C for 2 h, and then the supernatant was collected by centrifugation.
3. The supernatant obtained from the previous step was adjusted back to pH 6.0–7.0 using 5 M NaOH solution. Disodium hydrogen phosphate was added to a concentration of 10 mmol/L, monosodium dihydrogen phosphate to a concentration of 10 mmol/L, sodium chloride to a concentration of 0.15–1.0 mol/L, anhydrous sodium acetate to a concentration of 0.3–1.5 mol/L, and calcium chloride to a concentration of 0.25–0.5 mol/L. After being fully dissolved, acetic acid was added to adjust the pH to 5.40. Then, 20%–30% (v/v) of anhydrous ethanol was added according to the volume of the solution, thoroughly mixed, and left to stand at 2°C–8°C for 3–24 h before collecting the supernatant by centrifugation.
4. The supernatant obtained from the previous step was ultrafiltered using 100 kDa membrane to obtain a polysaccharide-enriched ultrafiltrate, the concentrated ultrafiltrate is then lyophilized and collected.

**FIGURE 1:**
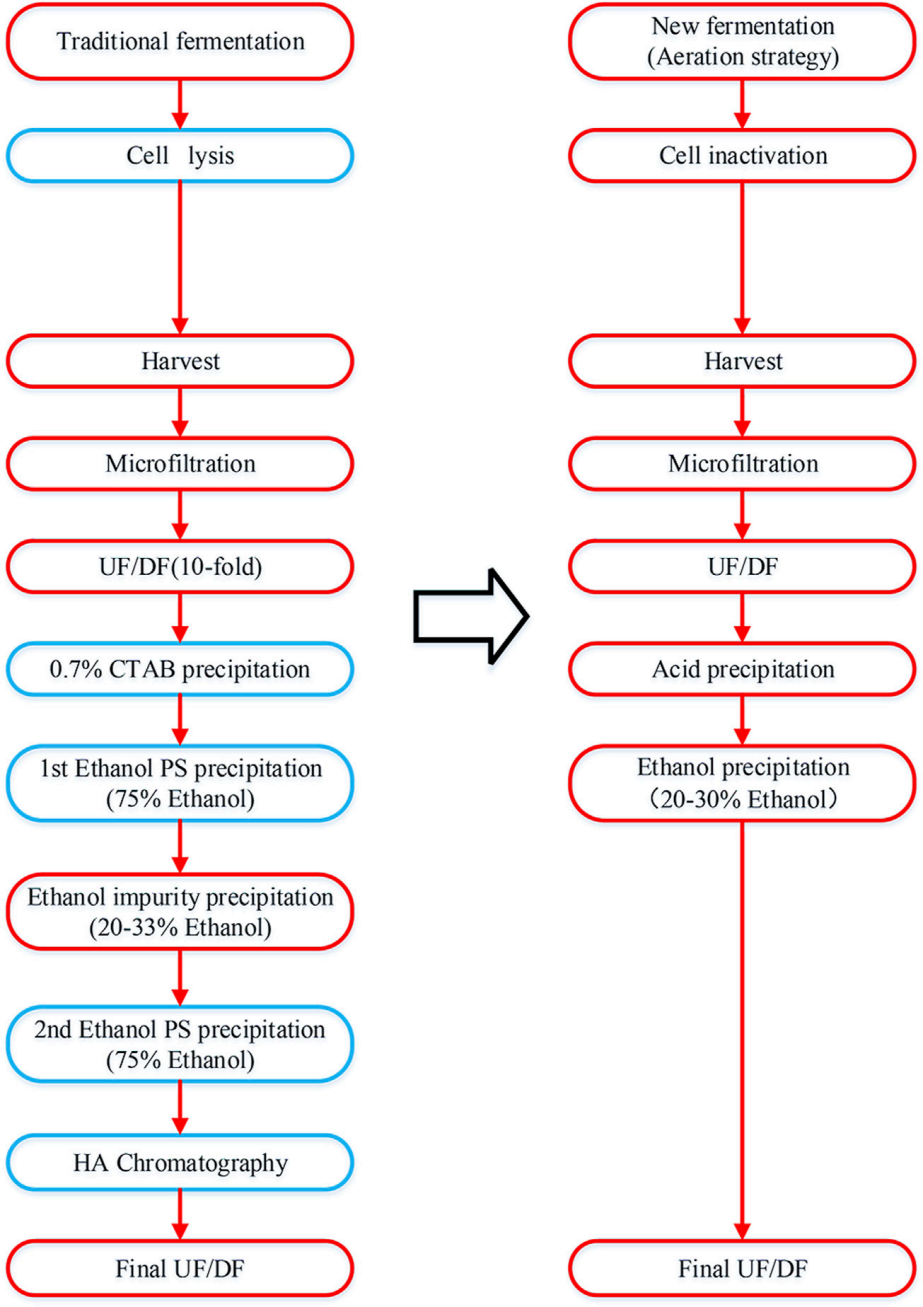
Process flowchart of pneumococcal polysaccharide purification. The traditional method is presented on the left and the simplified method in this study is presented on the right. The different purification steps are indicated in the blue box.

The comparison of purification steps for traditional method and this study is presented in Figure 1 (Lee et al., 2020).

### 2.6 Sample analysis

The content of polysaccharides in the supernatant and total polysaccharide of serotype 5 and serotype 10A were determined based on the rate nephelometric method using specific antisera obtained from Statens Serum Institute (Copenhagen, Denmark) according to immunochemical method (2.7.1) in the European Pharmacopoeia (11th edition); The purified CPSs from Type 5, 7F, 10A, 14, and 15B are subjected to tests according to European Pharmacopoeia (11th edition) including Protein (2.5.16), Nucleic acids (2.5.17), Total nitrogen (2.5.9), Phosphorus (2.5.18), Molecular size (2.2.30), Uronic acids (2.5.22), Hexosamines (2.5.20), Methyl pentoses (2.5.21), O-Acetyl groups (2.5.19). Protein was determined both by the Lowry method and modified Lowry method for serotype 5 polysaccharide.

## 3 Results and discussion

### 3.1 The release of polysaccharides into supernatant after switched aeration

As the phase variation (transparent and opaque) between two colony phenotypes of *S. pneumoniae* is a great versatility of the pneumococcus, the transparent variants have lower amounts of CPS and are more adapted for intranasal colonization (Weiser et al., 1994), whereas the opaque variants present higher amounts of CPS and are more adapted to survive in the bloodstream, as the capsule impairs opsonophagocytosis (Kim and Weiser, 1998). Therefore, the optimal strategy is to firstly culture *S. pneumoniae* in an environment which promotes the growth of opaque variant with large amount of CPS synthesis, after which the change of cultivation gas environment facilitates the transfer from opaque variants to transparent variants, allowing the exfoliating of CPS from pneumococcal cell wall with intact cell structure.

In this study, *S. pneumoniae* was cultured under completely anaerobic environment (100% carbon dioxide) during lag phase and exponential phase to promote bacterial growth to a high density as well as synthesis of CPS. In the late stage of exponential phase of bacterial growth, cultivation aeration was switched to 0.1 vvm air, which exerted environmental oxygen stress to facilitate the release of CPSs into culture medium. This aeration strategy was demonstrated to increase the release of CPS into supernatant for *S. pneumoniae* serotype 23F strain (Gonçalves et al., 2006). Therefore, in this study we aim to investigate the amount of soluble CPS in cultivation medium before and after the switch of cultivation atmospheric environment, which could potentially be purified with less complexity and lower impurity levels.

**FIGURE 2:**
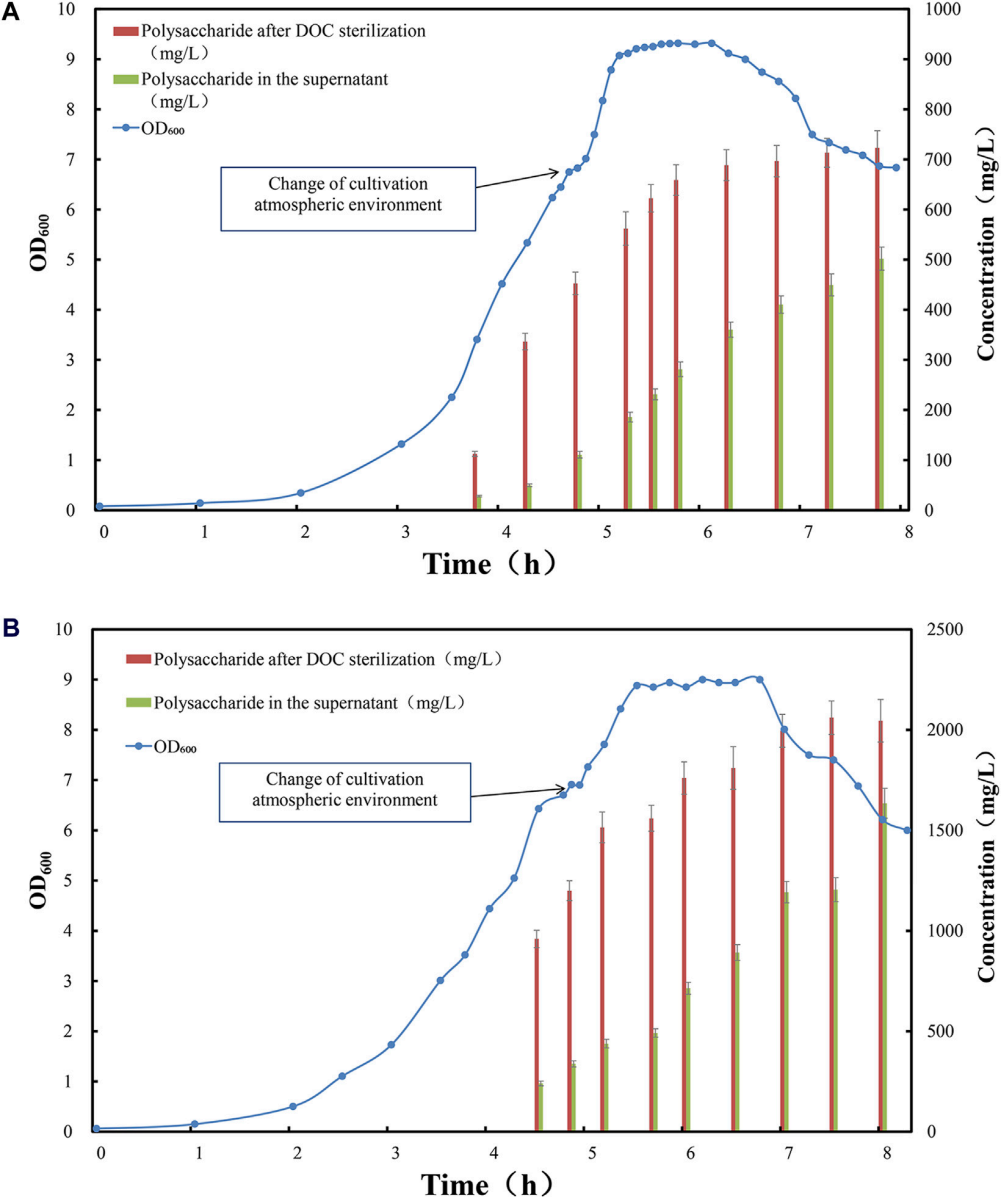
Time profile of *Streptococcus pneumoniae* serotype **(A)** 5 and **(B)** 10A fed-batch cultivation under CO_2_ environment, switched to air flow (0.1 vvm) in late exponential phase. Optical density measured at wavelength 600 nm is indicated by blue circle dots; the total amount of polysaccharide after complete cell lysis induced by DOC addition is indicated by red bar (mg/L); the amount of soluble polysaccharide in the fermentation supernatant is indicated by green bar (mg/L).

As serotype 5 and 10A CPS are generally purified with high residual protein level, therefore we firstly investigated the potential application of this novel aeration and inactivation strategy for the fermentation, as well as simplified purification process of serotype 5 and 10A polysaccharides. Lab scale fed-batch fermentation was carried out for serotype 5 and 10A, and the samples were taken from early stage of exponential phase until decline phase. The content of polysaccharide in supernatant and total polysaccharide were determined and demonstrated (Figure 2).

1. In the early and middle stages of the logarithmic growth phase of fermentation, the pneumococcal bacteria were grown in anaerobic environment with accumulation of biomass and high level CPSs.

**FIGURE 3:**
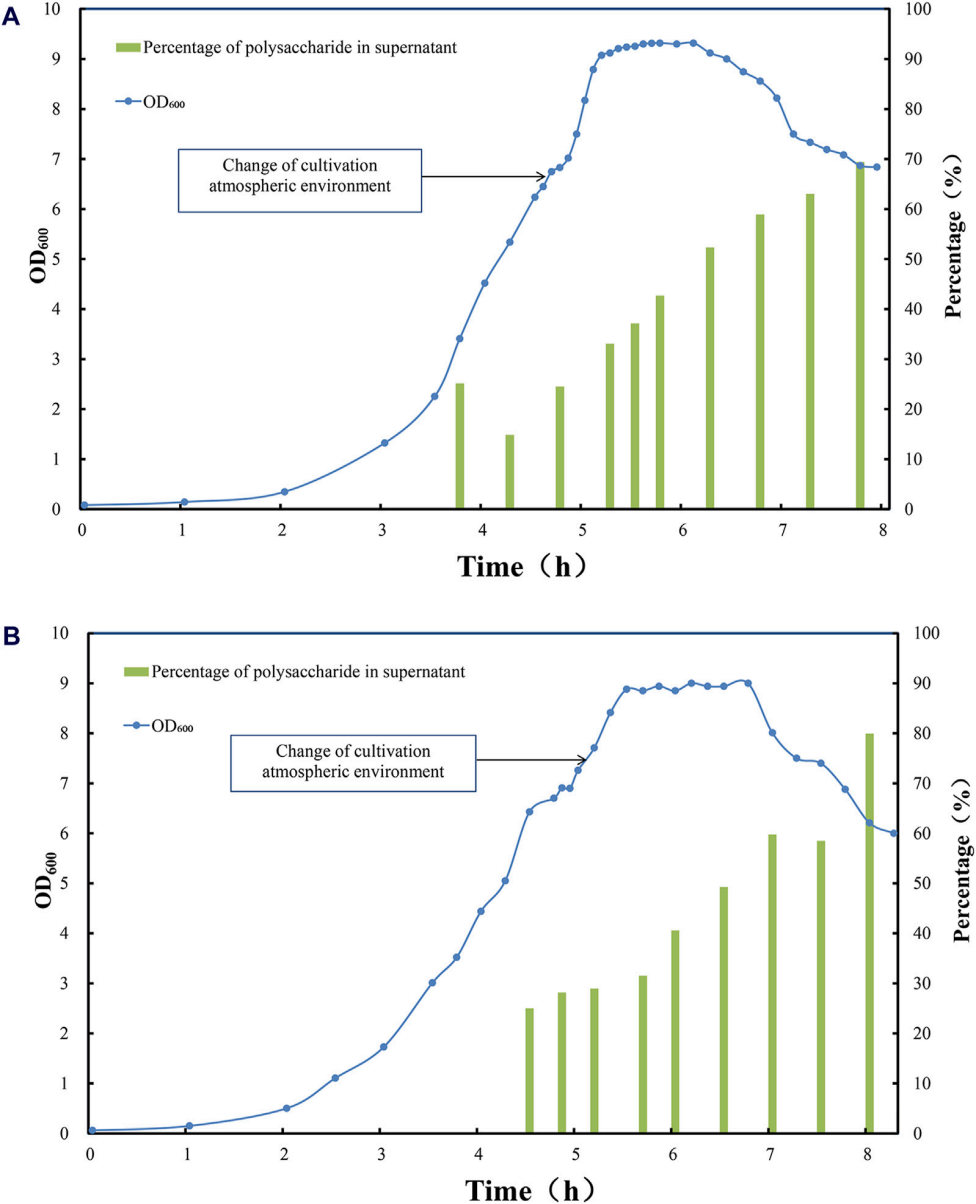
Time profile of *Streptococcus pneumoniae* serotype **(A)** 5 and **(B)** 10 A fed-batch cultivation under CO_2_ environment, switched to air flow (0.1 vvm) in late exponential phase. Optical density measured at wavelength 600 nm is indicated by blue circle dots; the percentage of soluble polysaccharide in total amount of polysaccharide after DOC cell lysis is indicated by green bar (%).

At this stage, the total amount of CPSs released by DOC lysis was low, among which the majority of polysaccharide was bound to the cell surface. For serotype 5, only 300–400 mg/L of polysaccharide was released after DOC cell lysis (Figure 2A), and approximately 80% of these was cell bound, leaving 20% CPS soluble in cultivation medium (Figure 3A); For serotype 10A, up to 1,000 mg/L of polysaccharide was released after DOC cell lysis (Figure 2B), and approximately 70%–80% of these was cell bound, leaving 20%–30% CPS soluble in cultivation medium (Figure 3B).

2. In the late stage of the logarithmic growth phase of fermentation, the cultivation gas environment of fermentation was adjusted from anaerobic 100% carbon dioxide to air, thereby inducing the phase transition of pneumococcal cells and promoting the release of CPSs.

During the logarithmic phase and the stationary phase, with the rapid growth of pneumococcal biomass and synthesis of CPS, the total amount of CPS after DOC cell lysis was increased to 700 mg/L for serotype 5 fermentation; due to the release from cell-bound CPS to supernatant, the soluble polysaccharide increased to 300–400 mg/L (Figure 2A), accounting for 40%–50% of total amount of CPS for serotype 5 (Figure 3A). Serotype 10A demonstrated a similar trend, the total amount of CPS after DOC cell lysis was increased to 1,700–1,800 mg/L; with the release of CPS from cell-bound fraction to supernatant, the soluble polysaccharide increased to 700–800 mg/L (Figure 2B), accounting for 40%–50% of total CPS (Figure 3B).

During this fermentation stage, biomass accumulation and polysaccharide biosynthesis slowed down, thus the total amount of CPS reached plateau, in the meantime the fraction of soluble polysaccharide in cultivation medium increased significantly due to this aeration strategy (Figure 3), with little intracellular components released to the medium. Shall a suitable sterilization method was applied to inactivate pneumococcal bacteria without damaging the overall integrity of the cells, not only a relatively high polysaccharide content could be recovered but also the lysis of pneumococcus cells could be avoided, which is conducive to simplifying downstream purification methods and reducing impurity residues.

3. At the end of the stationary phase and the decline phase, the autolysin secreted by pneumococcus began to split increasing fraction of pneumococcus cells, and pneumococcal biomass started to drop with little change in total CPS, which accompanied partial rupture of pneumococcus cells and release of increasing amount of CPSs, with soluble fraction of CPS in cultivation medium increased to 60%–70% for serotype 5% and 60%–80% for serotype 10A (Figure 3). However, the partial lysis of cells also released more intracellular impurities. Although the yield of polysaccharides has enhanced to a certain extent, it is not conducive to simplifying downstream purification.

### 3.2 Sterilization of fermentation broth by DOC, formaldehyde and BPL

Traditionally DOC is employed to sterilize pneumococcus since autolysin could be induced while sterilizing, provoking complete lysis of the pneumococcus cells and facilitating the release of all CPSs into cultivation medium, thereby achieving a high recovery of polysaccharides. This is also the reason why current mainstream process adopts DOC for sterilization. However, to facilitate downstream recovery and purification process of polysaccharide, two other inactivation reagents (formaldehyde or BPL) were also employed to sterilize fermentation broth during stationary phase after the switch of cultivation atmosphere in this study.

To confirm the inactivation efficiency of three methods (0.1% DOC, 0.1% BPL, 1% formaldehyde), the cell suspension after sterilization was plated on blood agar plates enriched with brain heart infusion, these plates were incubated in 10% CO_2_ at 36°C for up to 3 days and no colony forming units were observed for all three sterilization methods, which also complies with literature and prior knowledge (Gonçalves et al., 2002; Gonçalves et al. 2003; Gonçalves et al. 2006; Lu et al., 2010; Gogola et al., 2012; Gonçalves et al., 2014; Zanardo et al., 2016; Morais et al., 2019; Taddese et al., 2021).

**FIGURE 4:**
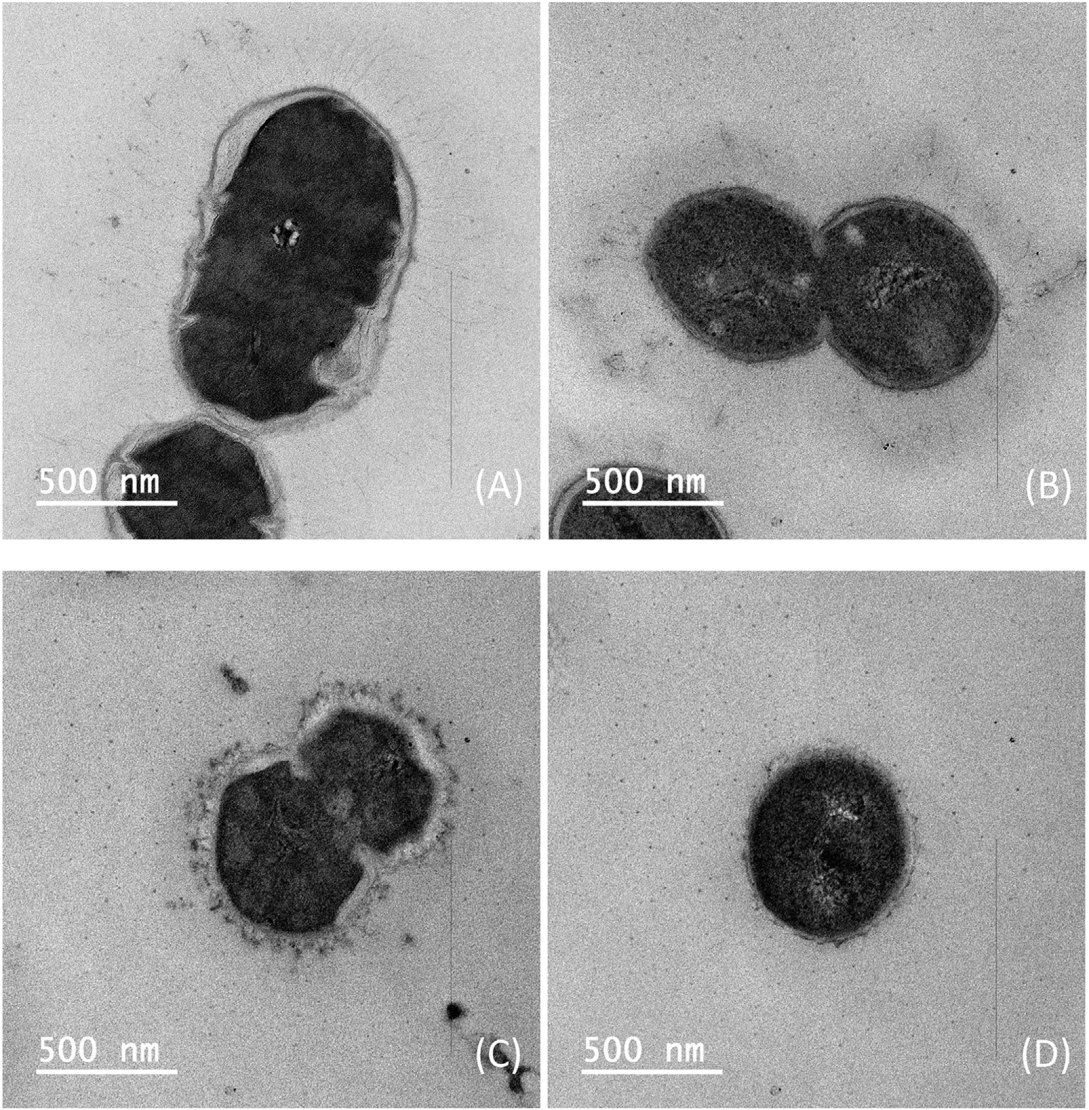
Transmission electron microscopy images of *Streptococcus pneumoniae* serotype 5 bacteria: **(A)** Inactivation by 1% formaldehyde, **(B)** Inactivation by 0.1% BPL; *Streptococcus pneumoniae* serotype 10A bacteria: **(C)** Inactivation by 1% formaldehyde, **(D)** Inactivation by 0.1% BPL (Size bar, 500 nm).

Since it is confirmed that three different sterilization methods can effectively inactivate Pneumococcus, to further determine the morphology of pneumococcal cells after sterilization using BPL and formaldehyde inactivation, cell suspension after sterilization was taken and observed under transmission electron microscope to examine the lysis of pneumococcal cells for serotype 5 and 10A. In contrast to traditional DOC sterilization, it can be observed from the TEM images that the pneumococcus fermentation broth sterilized with formaldehyde and BPL showed intact cell structures, indicating that the lysis of pneumococcus cells and the release of intracellular bacterial impurities were avoided during the sterilization process, which potentially facilitates the downstream purification process (Figure 4).

### 3.3 Quality of refined CPS after purification

Therefore, to further explore the beneficial effect of formaldehyde and BPL sterilization, a simplified purification process was applied to fermentation broth after sterilization to obtain purified capsular polysaccharide for tests. This purification method included ultrafiltration, acid precipitation, alcohol precipitation, final ultrafiltration and diafiltration, excluding traditional CTAB precipitation and phenol extraction, as well as the costly chromatography process (Figure 1).

Compared to the mainstream purification process, this simplified purification process only involves ultrafiltration and diafiltration to remove small molecular impurities, followed by acid and alcohol precipitation to eliminate protein and nucleic acid impurities, and finally diafiltration and lyophilization to obtain purified polysaccharide. This shortened purification process saves a significant amount of cost and time, purified polysaccharides for serotype 5, 7F, 10A, 14 and 15B were prepared from fermentation broth sterilized by three methods, and percentage contents of components for purified polysaccharides were analyzed according to European Pharmacopoeia (11th Edition) for comparison of test results (Table 1).

**TABLE 1:** Comparison of the yields and contents of components of serotype 5, 7F, 10A, 14, 15B bulk polysaccharide.

5	Inactivating agent	CPS yield (mg/L)	Protein	Nucleic acids	Total nitrogen (2.5%–6.0%)	Phosphorus (≤2%)	Molecular size (*K*_D_)	Uronic acid (≥12%)	Hexosamines (≥20%)
			(≤7.5%)	(≤2%)			CL-2B (≤0.60)		
	DOC	395 ± 32	5.9 ± 0.2	0.3 ± 0.1	4.5 ± 0.2	0.2 ± 0.1	0.39 ± 0.03	22.6 ± 0.5	27.1 ± 0.3
	BPL	271 ± 17	4.2 ± 0.1	0.2 ± 0.1	4.0 ± 0.1	0.3 ± 0.1	0.41 ± 0.01	19.7 ± 0.4	27.6 ± 1.2
	Formaldehyde	202 ± 12	4.2 ± 0.2	0.2 ± 0.1	4.1 ± 0.2	0.3 ± 0.1	0.36 ± 0.03	18.9 ± 0.7	27.2 ± 0.9
7F	Inactivating agent	CPS Yield (mg/L)	Protein	Nucleic acid	Total nitrogen (1.5%–4.0%)	Phosphorus (0%–1.0%)	Molecular size (*K*_D_)	Methyl pentose (≥13%)	
			(≤5%)	(≤2%)			CL-4B (≤0.20)		
	DOC	866 ± 25	1.2 ± 0.2	0.2 ± 0.1	2.5 ± 0.2	0.4 ± 0.1	0.01 ± 0.01	22.9 ± 0.8	
	BPL	614 ± 17	0.7 ± 0.2	0.1 ± 0.1	2.7 ± 0.2	0.5 ± 0.2	0.01 ± 0.01	23.5 ± 0.3	
	Formaldehyde	450 ± 35	0.6 ± 0.2	0.1 ± 0.1	2.5 ± 0.2	0.4 ± 0.1	0.02 ± 0.01	23.2 ± 0.4	
10 A	Inactivating agent	CPS Yield (mg/L)	Protein	Nucleic acid	Total nitrogen (0.5%–3.5%)	Phosphorus (1.5%–3.5%)	Molecular size (*K*_D_)	Hexosamines (≥12%)	
			(≤7%)	(≤2%)			CL-2B (≤0.65)		
	DOC	1,114 ± 84	3.4 ± 0.4	0.02 ± 0.01	1.3 ± 0.1	2.5 ± 0.1	0.42 ± 0.01	13.9 ± 0.3	
	BPL	734 ± 43	1.3 ± 0.2	0.01 ± 0.01	1.2 ± 0.2	2.4 ± 0.1	0.38 ± 0.02	14.1 ± 0.3	
	Formaldehyde	525 ± 39	1.0 ± 0.2	0.01 ± 0.01	1.3 ± 0.2	2.6 ± 0.1	0.41 ± 0.01	13.7 ± 0.7	
14	Inactivating agent	CPS Yield (mg/L)	Protein	Nucleic acid	Total nitrogen (1.5%–4.0%)	Phosphorus (0%–1.0%)	Molecular size (*K*_D_)	Hexosamines (≥20%)	
			(≤5%)	(≤2%)			CL-4B (≤0.30)		
	DOC	535 ± 18	1.4 ± 0.3	0.3 ± 0.1	2.4 ± 0.3	0.4 ± 0.1	0.10 ± 0.03	25.5 ± 0.5	
	BPL	381 ± 24	0.6 ± 0.1	0.2 ± 0.1	2.4 ± 0.2	0.5 ± 0.1	0.08 ± 0.03	25.8 ± 0.4	
	Formaldehyde	262 ± 13	0.5 ± 0.1	0.2 ± 0.1	2.3 ± 0.2	0.5 ± 0.2	0.08 ± 0.02	24.9 ± 0.3	
15B	Inactivating agent	CPS Yield (mg/L)	Protein	Nucleic acids	Total nitrogen (1.0%–3.0%)	Phosphorus (2.0%–4.5%)	Molecular size (*K*_D_)	Hexosamines (≥15%)	
			(≤3%)	(≤2%)			CL-2B (≤0.55)		
	DOC	711 ± 18	0.9 ± 0.1	0.05 ± 0.02	1.6 ± 0.2	3.1 ± 0.3	0.34 ± 0.02	17.7 ± 0.4	
	BPL	467 ± 12	0.6 ± 0.1	0.03 ± 0.02	1.5 ± 0.2	3.3 ± 0.2	0.32 ± 0.02	18.0 ± 0.4	
	Formaldehyde	351 ± 12	0.5 ± 0.1	0.02 ± 0.01	1.6 ± 0.2	3.2 ± 0.3	0.32 ± 0.02	17.3 ± 0.3	

**FIGURE 5:**
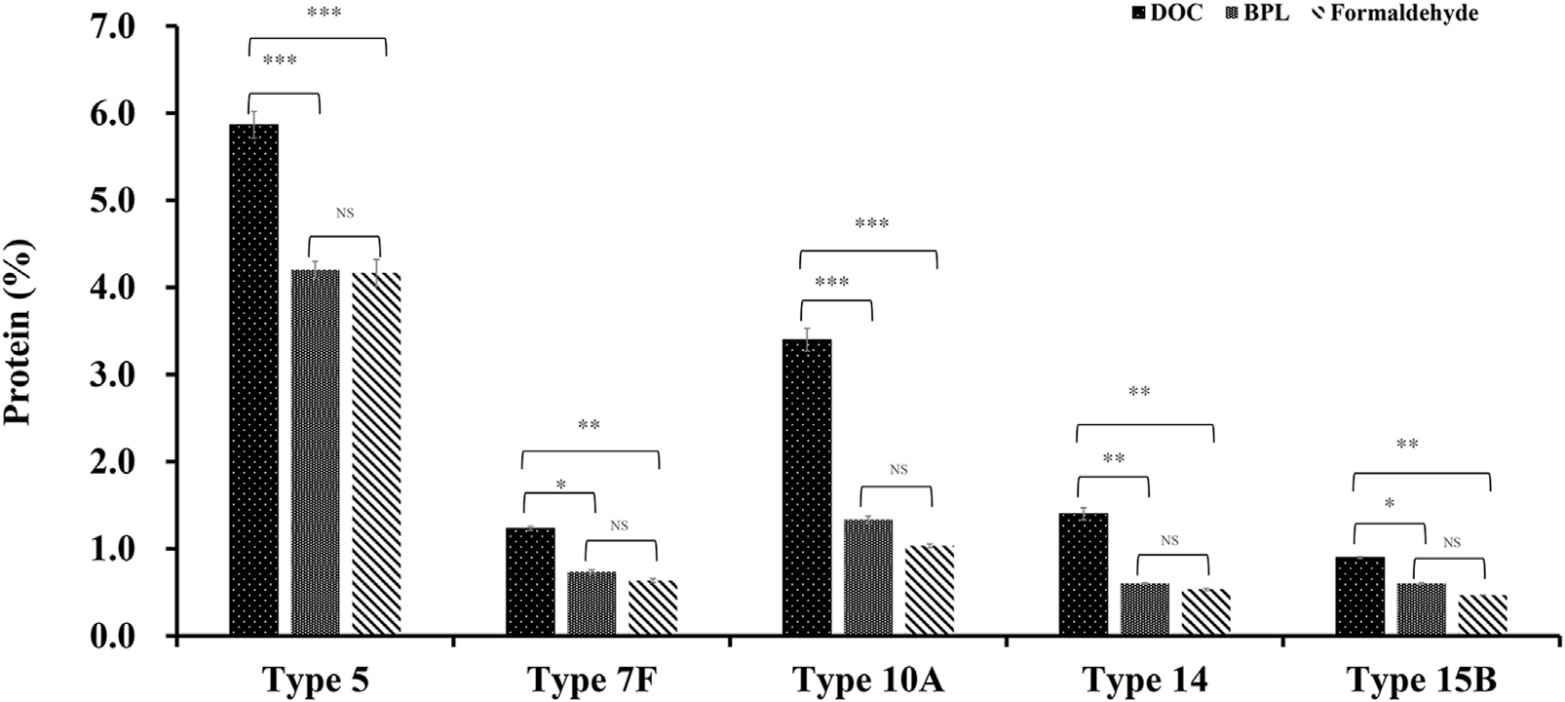
The comparison of protein impurity levels of refined CPS after three different inactivation methods (DOC, BPL and formaldehyde) for serotype 5, 7F, 10A, 14 and 15B (*p < 0.05, **p < 0.01, ***p < 0.001).

After comparison of traditional DOC sterilization with novel inactivation methods by BPL and formaldehyde, it was demonstrated that despite the decrease in CPS yield, residual protein concentration was significantly decreased from 5.9% to 4.2% for serotype 5, from 1.2% to 0.6% or 0.7% for 7F, from 3.4% to 1.3% or 1.0% for 10A, from 1.4% to 0.6% or 0.5% for 14, and from 0.9% to 0.6% or 0.5% for 15B (Figure 5), the levels of other contaminants such as nucleic acids were also slightly reduced after applying the improved processes. In the meantime, there is no obvious difference in impurity level between the purified polysaccharides obtained by BPL treatment and those obtained by formaldehyde treatment. The percentage contents of components were not affected by modification of the processes and could meet the quality control standards of European pharmacopoeia (11th Edition) (Table 1).

In summary, results showed that sterilization by formaldehyde and BPL rather than DOC could preserve intact cell and reduce intracellular contamination induced by cell lysis, resulting in much less impurity with simplified purification process, without affecting the quality of purified polysaccharide. This novel strategy was applied to 5 serotypes of CPSs in this investigation, the decreases in impurity level were consistently observed, indicating that the method is generally applicable. In particular, notable decreases were observed in serotypes 5, 10A and 14 (Figure 5).

### 3.4 Verification of scale-up process for serotype 5

**FIGURE 6:**
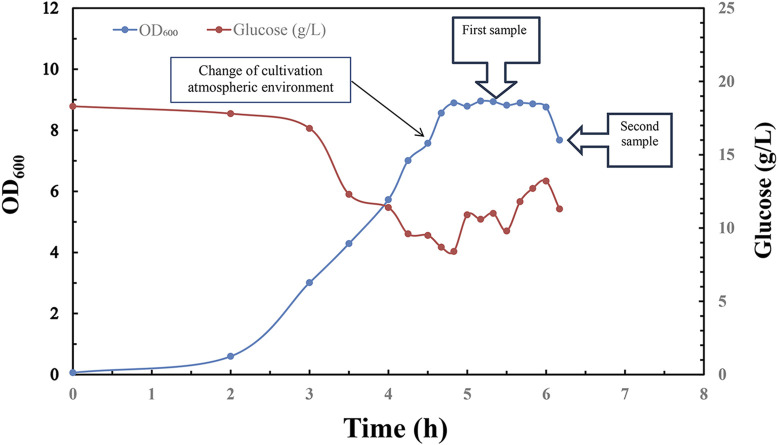
Time profile of *Streptococcus pneumoniae* 5 pilot scale fed-batch cultivation under CO_2_ environment, switched to air flow (0.1 vvm) in late exponential phase. Optical density measured at wavelength 600 nm is indicated by blue circle dots; Glucose concentration (g/L) in fermentation broth is indicated by red circle dots; 1st sample was taken in the middle of stationary phase for downstream process; 2nd sample was taken in the early stage of decline phase for downstream process.

In order to verify the scalability of this novel fermentation, sterilization and purification process for manufacturing of refined CPS, pilot scale experiment was conducted in 300 L bioreactor with a working volume of 200 L for serotype 5 *S*. *pneumoniae* (Figure 6).

Same aeration strategy was employed to facilitate the synthesis and release of pneumococcal CPS into culture medium during fermentation:

1. The first 100 L of fermentation broth sample was taken in the middle stage of stationary phase for sterilization with 0.1% DOC, 1% formaldehyde and 0.1% BPL respectively. After incubation at 4°C for 12 h, inactivated fermentation broth was further recovered and purified according to same purification protocol.
2. The remaining 100 L of fermentation broth was ceased in the early stage of decline phase, sterilized with 0.1% DOC, 1% formaldehyde and 0.1% BPL, respectively, further recovered and purified according to same protocol.

Pilot scale fermentation exhibited similar growth profile with a long stationary phase which is beneficial for inactivation operation in manufacturing process, and the pneumococcal optical intensity between small scale and pilot scale fermentation showed no obvious difference, and this suggested successful scaling up of fermentation process with this aeration strategy (Figure 6).

To investigate the repeatability of downstream process and quality of purified polysaccharide, the aforementioned samples were purified and tested according to the same purification protocol. This table with the percentage content of components of serotype 5 refined polysaccharides demonstrated potential effects of the inactivation time on the quality of refined polysaccharide.

The 1^st^ sample was taken in the middle of stationary phase when no obvious cell lysis took place, and test results showed that the residual protein contents in the refined polysaccharides obtained after BPL and formaldehyde sterilization are 4.4% and 4.2%, respectively, similar to the results achieved with lab-scale experiment. In the meantime, BPL inactivation achieved higher yield, released 446 mg/L to the supernatant, accounting for 67% of total CPS (DOC inactivation), in contrast, formaldehyde inactivation achieved lower recovery yield, with 321 mg/L in supernatant. All the other test results demonstrated good consistency with small scale results (Table 2).

**TABLE 2:** Comparison of the yields and contents of components of serotype 5 bulk polysaccharide.

	Inactivating agent	CPS yield (mg/L)	Polysaccharide in supernatant (mg/L)	Percentage (%)	Protein by lowry method (%)	Protein by modified lowry method (%)	Nucleic acids (%)	Total nitrogen (%)	Phosphorus (%)	Molecular size (CL-2B K_D_≤0.60)	Uronic acid (%)	Hexosamines (%)
1st sample	DOC	386	669	100	5.8	0.9	0.3	4.2	0.3	0.38	22.1	26.4
	Formaldehyde	178	321	48	4.2	0.4	0.2	4.4	0.2	0.40	21.5	26.1
	BPL	242	446	67	4.4	0.5	0.2	4.3	0.3	0.39	21.8	25.2
2nd sample	DOC	408	712	100	6.0	0.9	0.3	4.3	0.3	0.39	22.8	25.4
	Formaldehyde	214	384	54	4.4	0.5	0.2	4.1	0.4	0.42	21.4	24.7
	BPL	278	512	72	4.6	0.5	0.2	4.5	0.3	0.41	20.6	25.9

The 2nd sample for purification was taken in the early stage of decline phase, during which the secretion of autolysin was initiated and partial lysis of pneumococcal bacteria released both target polysaccharide as well as intracellular impurities. The test results for 2nd sample showed that the residual protein content in the refined polysaccharides obtained after BPL and formaldehyde sterilization are 4.6% and 4.4%, slightly higher compared to the results achieved with 1st sample, all the other test results demonstrated good consistency with small scale results. In addition, both BPL and formaldehyde inactivation achieved slightly higher yield compared with 1st sample, BPL inactivation released 512 mg/L to the supernatant, accounting for 72% of total CPS (DOC inactivation); formaldehyde inactivation released 384 mg/L in supernatant, accounting for 54% of total CPS. All the other test results demonstrated good consistency with small scale results (Table 2).

Therefore, it can be concluded that sterilization with BPL and formaldehyde during stationary phase could maintain overall integrity of the cells and avoid the lysis of pneumococcus cells, which is conducive to simplifying downstream purification methods and reducing impurity levels in purified polysaccharide. In the meantime, sterilization with BPL and formaldehyde in decline phase could release polysaccharide accounting for 54%–72% of total CPS, but caused slightly increased level of impurities in purified polysaccharide. In addition, sterilization with BPL could release a higher percentage of total polysaccharide although its impurity level is also slightly higher than formaldehyde sterilization, this phenomenon was also supported by comparing the thickness of capsules in TEM microscopy after sterilization with formaldehyde and BPL (Figure 4).

## 4 Conclusion

CPSs as efficient antigens in pneumococcal vaccines, must meet strict quality requirements to ensure sufficient safety. Quality control requirements related to vaccine safety include the control of residual bacterial nucleic acids, proteins, pyrogenic substances and the content of chemicals related to purification. Traditionally, in the final stage of fermentation, the addition of DOC or other lysis agents to the fermentation broth inactivates the pneumococcus while the autolysin produced by the pneumococcus promotes the lysis of the bacterial cells, resulting in the release of a large amount of cellular debris, including proteins, nucleic acids, cell wall components and other impurities. Therefore, this method of inactivation is unfavorable for subsequent purification, and complex purification process of CPS is necessary for the removal of impurities.

This paper explores a novel fermentation method that firstly culture *S*. *pneumoniae* under anaerobic environment and alters the culture gas environment at the end of the logarithmic growth phase, promoting the release of CPSs from the surface of pneumococcus into the supernatant. Secondly, BPL and formaldehyde were employed for inactivation, which avoided the lysis of pneumococcus cells during the sterilization process of the fermentation broth, BPL inactivation also improved the extent of CPS release. Finally, simplified downstream purification process was performed on inactivated fermentation broth, which included only ultrafiltration, acid precipitation, and ethanol precipitation to obtain refined polysaccharide with lower impurity levels. The refined polysaccharides obtained from 5, 7F, 10A, 14, and 15B were subjected to a variety of tests, results showed that application of this novel aeration strategy and inactivation methods by formaldehyde and BPL in fermentation process, as well as simplified purification method could lead to refined polysaccharides meeting European pharmacopoeia standards with lower impurities. Furthermore, pilot scale fermentation, inactivation and purification production process were carried out to verify the scalability of this novel polysaccharide manufacturing process.

The research data strongly validates the feasibility of using BPL and formaldehyde sterilization as alternatives to DOC sterilization methods. Although there is a disadvantage in terms of the release of polysaccharide, this limitation could be compensated by improving fermentation process, such as culture medium formulation, fermentation process parameters and optimization of the timing of fermentation harvest. In addition, the simplification of downstream process is also beneficial for the increase of recovery yield, reduction in cost and duration of purification process. As the valency of pneumococcal vaccines increases, the accumulation of protein and nucleic acid impurities in purified polysaccharides also rises. The selection of novel sterilization methods such as BPL sterilization can provide theoretical basis and technical guidance for controlling impurities during the purification process of higher-valency pneumococcal polysaccharide vaccines and pneumococcal conjugate vaccines.

## Data Availability

The raw data supporting the conclusions of this article will be made available by the authors, without undue reservation.
